# ANALYSIS OF REST-ACTIVITY RHYTHMS AND RISK OF INCIDENT MILD COGNITIVE IMPAIRMENT AND DEMENTIA IN OLDER WOMEN

**DOI:** 10.1093/geroni/igz038.1496

**Published:** 2019-11-08

**Authors:** Katie L Stone, Katie L Stone, Terri Blackwell, Jamie Zeitzer, Kristine Yaffe, Sonia Ancoli-Israel, Susan Redline, Gregory J Tranah

**Affiliations:** 1 California Pacific Medical Center Research Institute, San Francisco, California, United States; 2 California Pacific Medical Center, San Francisco, California, United States; 3 Stanford University, Palo Alto, California, United States; 4 University of California, San Francisco, San Francisco, California, United States; 5 University of California, San Diego, San Diego, California, United States; 6 Brigham and Women’s Hospital, Boston, Massachusetts, United States; 7 Sutter Health, San Francisco, California, United States

## Abstract

In older adults, desynchronized circadian rhythms have been associated with medical illness, including Alzheimer Disease. Activity, which can be easily measured using actigraphy over consecutive 24-hour periods, is a valid marker of entrained sleep phase and correlates with entrained endogenous circadian phase. We compare results of both parametric and non-parametric analyses to test the association of rest-activity patterns with incident MCI and dementia in 2132 older women who had 2 or more 24-hrs periods of actigraphy data collected at baseline. Follow-up neuropsychological testing approximately 5 years later is used to classify women as normal, MCI, or dementia. Logistic regression models are adjusted for age, clinic site, race, education, body mass index, functional status, comorbidities, medication use, and health habits. Results suggest the importance of overall amplitude and rhythmicity, as well as timing of activity patterns over the 24-hour day as risk factors for incident MCI/dementia.

